# Physiological effects of sodium bicarbonate ingestion via Maurten Bicarb on endurance running performance

**DOI:** 10.1080/15502783.2025.2550173

**Published:** 2025-09-08

**Authors:** Lukas Keverkamp, Kelsey Scanlon

**Affiliations:** Walsh University, Department of Exercise Science, North Canton, OH, USA

**Keywords:** Supplement, GI response, endurance, performance

## Abstract

**Introduction:**

Extensive research has examined the effectiveness of sodium bicarbonate as an acid-base buffer for enhancing exercise performance; however, limited research at this time has focused specifically on endurance runners or the Maurten Bicarb System. This study aimed to examine the effects of the Maurten Bicarb System, a sodium bicarbonate-based supplement, on both performance and gastrointestinal (GI) responses in endurance runners.

**Methods:**

Eight collegiate endurance athletes (four male, four female; aged 19–22 years old) from a Division II Cross Country and/or Track and Field team participated in this study. Each participant completed two treadmill interval running trials at a pace 10 s per mile faster than their predicted lactate threshold to approximate anaerobic threshold. Prior to one trial, participants consumed the Maurten Bicarb System according to manufacturer instructions. GI symptoms were assessed via the GI Symptom Experience Survey (GISES) before and after both trials. Additionally, respiratory exchange ratio (RER), rate of perceived exertion (RPE), heart rate (HR), and blood lactate (BL) were measured after each running interval. A one-way repeated measures ANOVA was used to analyze changes in performance variables, while a two-way ANOVA assessed the main effects of time and condition on GI symptoms.

**Results:**

The results showed no significant differences (*p* > 0.05) in RER, RPE, HR, or BL between the two trials, indicating no measurable improvement in performance. Similarly, there was no significant change (*p* > 0.05) in GI symptoms from pre- to post-exercise in either trial condition.

**Conclusion:**

The Maurten Bicarb System did not significantly enhance performance in this sample of endurance runners but notably avoided the gastrointestinal distress that typically accompanies sodium bicarbonate ingestion. These findings highlight the need for further research, potentially exploring different exercise intensities, durations, or physiological markers, to better determine the efficacy of sodium bicarbonate supplementation in endurance performance.

